# Pathogenic Role and Antibiotic Resistance of Methicillin-Resistant *Staphylococcus aureus* (MRSA) Strains Causing Severe Community-Acquired Pneumonia in Vietnamese Children

**DOI:** 10.3390/arm91020012

**Published:** 2023-03-30

**Authors:** Khai Quang Tran, Thuy Thi Dieu Nguyen, Van Hung Pham, Quan Minh Pham, Hung Do Tran

**Affiliations:** 1 Department of Pediatrics, Can Tho University of Medicine and Pharmacy, Can Tho City 90000, Vietnam; 2 Department of Pediatrics, Hanoi Medical University, Hanoi 100000, Vietnam; 3 Laboratory of Nam Khoa Biotek Company, International Research of Gene and Immunology Institute, Ho Chi Minh City 700000, Vietnam; 4 Department of Nursing and Medical Technology, Can Tho University of Medicine and Pharmacy, Can Tho City 90000, Vietnam

**Keywords:** methicillin-resistant *Staphylococcus aureus*, antibiotic, resistance, community-acquired pneumonia, children

## Abstract

**Highlights:**

**What are the main findings?**

- Methicillin-resistant *Staphylococcus aureus* (MRSA) plays a greatly important role as the second leading cause of severe community-acquired pneumonia (CAP) in Vietnamese children;
- All isolates of MRSA are resistant to many antibiotics and sensitive to vancomycin and linezolid.

**What are the implications of the main finding?**

- MRSA agents should be considered for empiric antibiotic therapy for severe CAP in children because of the role of MRSA in the disease;
- It is necessary to have a rational antibiotic use strategy to prevent vancomycin resistance in the future.

**Abstract:**

In recent years, the pathogenic role and antibiotic resistance of methicillin-resistant *Staphylococcus aureus* (MRSA) strains causing severe community-acquired pneumonia (CAP) have received increasing attention in clinical practice. The aim of this study was to determine the rate of isolates of MRSA strains causing severe CAP in children and to assess their level of antibiotic resistance. The study design was cross-sectional. Children with severe CAP were sampled by nasopharyngeal aspiration for the culture, isolation, and identification of MRSA. Antimicrobial susceptibility testing was performed using the gradient diffusion method to determine the minimum inhibitory concentration (MIC) of antibiotics. Results: MRSA was identified as the second leading cause of severe CAP in Vietnamese children. The rate of isolates of *S. aureus* was 41/239 (17.5%), of which most were MRSA, at 32/41 (78.0%). MRSA strains were completely non-susceptible to penicillin (100%), more resistant to clindamycin and erythromycin, less sensitive to ciprofloxacin and levofloxacin, and fully susceptible to vancomycin and linezolid, with a 32-fold decreased MIC_90_ for vancomycin (0.5 mg/L) and a 2-fold decreased MIC_90_ for linezolid (4 mg/L). Therefore, vancomycin and linezolid may be appropriate options for severe CAP identified by MRSA.

## 1. Introduction

In recent years, methicillin-resistant *Staphylococcus aureus* (MRSA) has received increasing attention in clinical practice in many countries around the world [1,2,3]. Previously, MRSA was known to play a major role in skin and soft tissue infections [4]; however, it has been increasingly associated with invasive infections, especially severe community-acquired pneumonia (CAP) in children, with an increasing prevalence worldwide [2,5].

MRSA is defined as a strain of *S. aureus* with a minimum inhibitory concentration (MIC) of oxacillin ≥ 4 mcg/mL [6]. MRSA is primarily mediated by the *mecA* gene encoding the penicillin-binding protein 2a. Today, because cefoxitin is a strong inducer of *mecA*, it is widely used to replace the antibiotic oxacillin in the detection of MRSA strains via the *mecA* gene. Therefore, cefoxitin-resistant strains of *S. aureus* are considered MRSA with an MIC threshold of cefoxitin ≥ 8 mcg/mL [6]. MRSA is resistant to nearly all beta-lactam antibiotics and is often resistant to antibiotics of other classes, such as macrolides, aminoglycosides, glycopeptides, and lipopeptides [7,8]. This becomes a major threat to human health in the context of infectious diseases and a great challenge for clinicians in regard to the use of therapeutic antibiotics. In fact, the trend of multi-antibiotic resistance has been increasing in recent years. Data on children with MRSA infection and the antibiotic resistance of isolates from 11 children’s hospitals in China noted that MRSA isolates are highly resistant to erythromycin (76.88%) and clindamycin (54.97%) and 100% resistant to penicillin [9].

In Vietnam, the prescription of antibiotics upon hospital admission was found to be mainly based on local guidelines (62.3%), drugs used before hospital admission (50.0%), and the opinions of senior clinicians (up to 37.7%) [10]. A recent study conducted on 2911 children hospitalized for pneumonia in a large city in Vietnam showed that 89.2% of children with non-severe pneumonia required intravenous antibiotics [11]. This shows that there is a need to study the antibiotic resistance of common pathogens, thereby creating a more effective antibiotic management strategy.

Until now, no study has been conducted on the antibiotic resistance level of *S. aureus*, as well as MRSA causing severe CAP in children. We performed such a study at Can Tho Children’s Hospital, which is the largest pediatric hospital in the Mekong Delta, South Vietnam. The current treatment is mainly based on documents from other regions or countries. The purpose of this study was to determine the rate of isolates of *S. aureus* and MRSA strains causing severe CAP in children at Can Tho Children’s Hospital, Vietnam, and assess the level of antibiotic resistance through MIC determination. This study’s findings will serve as an extremely important database for more targeted and effective antibiotic treatments.

## 2. Materials and Methods

### 2.1. Subjects

The study was carried out on 239 children with severe CAP hospitalized at Can Tho Children’s Hospital, Vietnam, from March 2020 to February 2021.

The inclusion criteria were children aged from 2 months to 15 years with pneumonia who had been admitted to the hospital within the first 48 h of infection. Pneumonia was clinically diagnosed according to the World Health Organization (WHO) criteria [12], as cough or dyspnea with at least one of the following signs: tachypnea by age, chest retraction, or an abnormal lung examination result, including hypoventilation and pulmonary rales. Severity was assessed according to the British Thoracic Society (BTS) criteria [13], with at least one of the following signs: fever > 38.5 °C, respiratory rate > 70 breaths/minute (infants), >50 breaths/minute (older children), moderate/severe intercostal muscle contractions, nasal flaring, grunting, apnea, cyanosis, lack of feeding (infants) or dehydration (older children), capillary refill time ≥ 2 s, or peripheral blood oxygen saturation SpO_2_ < 92%. All cases were confirmed as pneumonia on chest X-ray.

The exclusion criteria were patients who had been hospitalized within 14 days before the onset of symptoms; cases where the sample was collected before this timeframe; pneumonia caused by inhalation, aspiration, or drowning; or children whose families refused to join the study group. There were not any children with chronic respiratory diseases or immune disorders participating in this study.

### 2.2. Study Design

The study design was cross-sectional. All hospitalized children who met the inclusion criteria were invited to participate in the study group. First, the patients were investigated to acquire some general information, followed by a clinical examination and checks for the white blood cell (WBC) count using a SIEMENS ADVIA^®^ 2120i hematology analyzer (Siemens Healthineers, Erlangen, Germany) and C-reactive protein (CRP) using an AU480 biochemical analyzer (Beckman Coulter, Brea, California (CA), USA). Simultaneously, nasotracheal aspiration (NTA) samples were collected from all patients using a special mucus extractor device (Global Medikit Limited, New Delhi, India). Then, the samples were transported to the International Research of Gene and Immunology Institute, Laboratory of Nam Khoa Biotek Company, Ho Chi Minh City, Vietnam, a laboratory that meets the ISO 9001:2015 13485:2017 and WHO-GMP (TRS 908, ANNEX 4) standards, so as to implement the screening, culture, and antimicrobial susceptibility testing. In the laboratory, the samples were quality checked before processing, confirming that they had been obtained from the lower respiratory tract. This was based on an assessment of the number of squamous epithelial cells (SECs) and polymorphonuclear cells (PMNs) in a Gram stain smear of the specimen. The presence of <10 SECs and >25 PMNs per low-power field (magnification, x100) was regarded as indicative of a high-quality expectorated sputum specimen [14,15].

**Figure 1:**
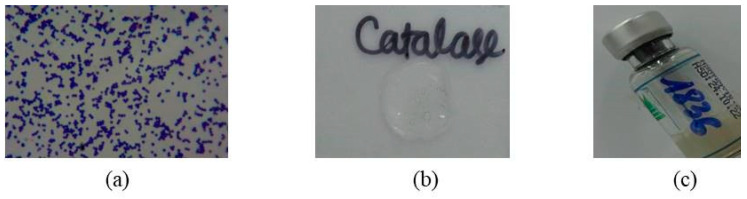
Result of culture isolates of *Staphylococcus aureus* from a boy aged 7 months old in this study. (**a**). Gram (+) cocci staining; (**b**). Catalase (+); (**c**). Coagulase (+).

*S. aureus* was isolated through Gram cocci (+) staining, catalase (+), and coagulase (+) reactions (Figure 1).

MRSA strains were identified when cefoxitin MIC ≥ 8 mcg/mL [6].

Antimicrobial susceptibility testing (AST) was performed using the gradient diffusion method. Strips from the Nam Khoa Company, Vietnam, infused with a predefined gradient of antibiotic concentrations, were used to determine the MICs of antibiotics including penicillin, clindamycin, erythromycin, gentamicin, ciprofloxacin, levofloxacin, chloramphenicol, vancomycin, linezolid, and rifampin. The MIC breakpoint used to determine the susceptible, intermediate, and resistantvalues of the antibiotics was applied in accordance with the guidelines of the manufacturer, Nam Khoa, and the Clinical and Laboratory Standards Institute (CLSI) for 2021 [6]. MIC_50_ and MIC_90_ are concentrations at which 50% and 90% of the bacterial strains are inhibited, respectively [6].

### 2.3. Statistical Analyses

The data were analyzed using Statistical Package for Social Sciences (SPSS) software version 18.0 (International Business Machines Inc., Armonk, NY, USA). The steps of this method were as follows: First, calculate frequencies and percentages for the qualitative variables. Second, calculate the mean (or median) and standard deviation for the quantitative variables. Third, compare and identify the difference between the two ratios based on the Chi-square test. Finally, compare the mean/median of the 2 groups based on the *t*-test (normally distributed) and U-test (not normally distributed). The multi-group mean/median comparisons were based on the ANOVA test (normally distributed) and the Kruskal–Wallis test (not normally distributed). A value of *p* < 0.05 was determined to be statistically significant. Next, we analyzed the nutritional indicators using the WHO Anthro Survey Analyzer software, version 3.2.2, (http://www.who.int/childgrowth/software/en/, accessed on 2 May 2021) for children < 5 years old and Anthroplus Survey Analyzer software, version 1.0.4, (http://www.who.int/tools/growth-reference-data-for-5to19-years/application-tools, accessed on 2 May 2021) for children ≥ 5 years old.

### 2.4. Ethics Approval

This study was approved by the Institutional Review Board (IRB) for Ethics Committee in Biomedical Research of Hanoi Medical University, Hanoi, Vietnam (No. 89/GCN-HĐĐĐNCYSH-ĐHYHN).

## 3. Results

### 3.1. Isolation Rate and Demographic, Clinical, and Subclinical Characteristics

**Figure 2:**
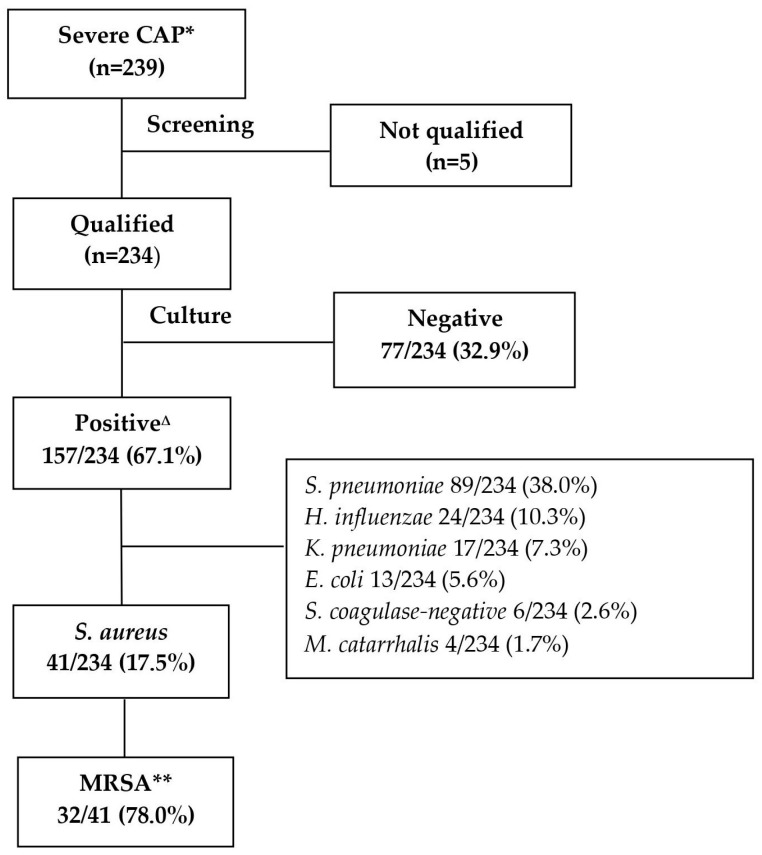
Research flowchart. * CAP: community-acquired pneumonia; ** MRSA: Methicillin-resistant *Staphylococcus aureus*; ^∆^: Among the 157 positive cases, there were 37 co-infections with microorganisms, including 14 co-infections with *S. pneumoniae* and *H. influenzae*, 7 co-infections with *S. pneumoniae* and *S. aureus*, 5 co-infections with *S. pneumoniae* and *K. pneumoniae*, 4 co-infections with *S. aureus* and *K. pneumoniae*, 2 co-infections with *S. pneumoniae* and *S. coagulase-negative*, 2 co-infections with *S. pneumoniae* and *E. coli*, 2 co-infections with *K. pneumoniae* and *H. influenzae*, and 1 co-infection with *S. aureus* and *E. coli*.

A total of 239 children with severe CAP admitted to Can Tho Children’s Hospital provided NTA samples. There were five samples that did not meet the standards for lower respiratory tract specimens, with a result of >10 SECs and <25 PMNs, so that culture could not be performed. The remaining 234 samples qualified for culture. The positive culture rate was 157/234 (67.1%). Through bacterial isolation culture, we found that *Streptococcus pneumoniae* had the highest isolation rate, at 89/234 (38.0%), followed by *S. aureus* at 41/234 (17.5%), of which most were MRSA, at 32/41 (78.0%) (Figure 2).

The median age of children with severe CAP caused by MRSA was 14 months. The disease was most common in children under 2 years old (75.0%), followed by those aged 2–5 years old (21.9%), and less common in children over 5 years old (3.1%). Boys were more susceptible than girls, with a male–female sex ratio of 3.6:1. All children with severe CAP had a fever and cough. Compared with methicillin-sensitive *Staphylococcus aureus* (MSSA), MRSA tended to have more symptoms, such as tachypnea, chest indrawing, contractions of the accessory respiratory muscles, crackles, wheezing, and hypoxemia (SpO_2_ ≤ 94%) in children with severe CAP. However, the difference was not statistically significant (except for signs of accessory respiratory muscle contraction, with *p* = 0.04). The mean WBC count was 13.56 ± 6.44 (×10^3^/mm^3^). Compared with other etiologies, MRSA was less likely to cause leukocytosis >15,000/mm^3^ (*p* = 0.048). CRP had a median value of 12.5 mg/L, while the lowest was 1.1 mg/L and the highest was 255 mg/L. In total, 58.1% of cases had an increase in CRP > 10 mg/L (Table 1).

### 3.2. Antibiotic Resistance and Minimum Inhibitory Concentration

The MRSA isolates were completely resistant to penicillin; resistant to many antibiotics such as clindamycin (84.4%), erythromycin (78.1%), and gentamicin (56.3%); and fully sensitive to vancomycin and linezolid. Compared with MSSA, the MRSA strains were more resistant to clindamycin and erythromycin and less sensitive to ciprofloxacin and levofloxacin (*p* < 0.05) (Table 2).

Penicillin, erythromycin, clindamycin, gentamicin, ciprofloxacin, levofloxacin, and chloramphenicol had an MIC_90_ equal to the resistance threshold according to the CLSI guidelines of 2021. Vancomycin, linezolid, and rifampin had an MIC_90_ lower than the CLSI threshold of resistance, of which vancomycin had a 32-fold decreased MIC_90_ (0.5 mg/L) and linezolid had a 2-fold decreased MIC_90_ (4 mg/L) (Table 3).

## 4. Discussion

### 4.1. Isolation Rate and Demographic, Clinical, and Subclinical Characteristics

Until now, *S. pneumoniae* and *Haemophilus influenzae* type b (Hib) had always been the dominant agents causing CAP in children [16,17]. However, in recent years, the increased use of pneumococcal conjugate vaccines and Hib vaccines in many countries around the world has changed the cause of pneumonia. Many studies have shown that the rates of *S. pneumoniae* and Hib have been significantly reduced in countries with good immunization backgrounds, while *S. aureus* and *H. influenzae* non-type b are currently the most common pathogens [15,18]. In this study, *S. aureus* was identified as the second leading organism isolated from the NTA of children with severe CAP, as observed in 41/234 cases (17.5%), of which most were MRSA, at 32/41 (78.0%). This result is quite similar to that of a study conducted by Doudoulakakis AG et al. in Greece, published in 2016, in which 41/132 (31.06%) cases were *S. aureus*, while MRSA accounted for 31/41 (75.6%) [19]. MRSA was also recorded at a very high rate in the study of Ensinck G et al. conducted in Argentina (85%) [2]. The pathogenic role of MRSA in severe CAP in children is increasingly being demonstrated in many countries. Empiric antibiotics should cover the causative agent of MRSA.

Severe CAP caused by MRSA was most commonly observed in children under 2 years old (75.0%), followed by those aged 2–5 years old (21.9%), and less common in children over 5 years old (3.1%). This result is consistent with the literature and many studies which noted that children under 5 years of age, especially infants under 12 months old, are more susceptible to pneumonia than older children and more likely to develop more severe illness [16,20]. This is explained because children under 12 months of age suffer from a decrease in passive immunity from the mother, while the body’s active immunity has not yet fully formed [21]. Gender is an important factor influencing morbidity and mortality in children, but its role in general respiratory infections in children remains unclear [22]. However, there have been many previous studies that found the male–female sex ratio in children with pneumonia is usually >1/1, and this rate increases even more for severe cases [23,24,25]. Doudoulakakis AG et al. recorded a rate of up to 60.9% of boys with CAP due to *S. aureus*, while Frush JM et al. recorded this rate as 74% [19,26]. This study found that boys tend to be more susceptible than girls, with a male–female sex ratio of 3.6/1. This significant difference may be due to the fact that this study was conducted on children with severe pneumonia.

All children with severe CAP had a fever and cough. In a multicenter study in the United States that included 2358 children < 18 years of age who were hospitalized with evidence of pneumonia upon chest radiograph, 95% of patients had a cough and 90% of patients had a fever [23]. Coughs and fevers are highly sensitive in CAP, especially severe CAP. However, the specificity of coughs and fevers is not high in pneumonia. According to Shah SN et al., fever had a sensitivity of 80–92% in pneumonia but a specificity of only 47–54% [27]. In this study, the common clinical symptoms of children with MRSA-related severe CAP were tachypnea (93.8%), crackles (87.5%), and chest indrawing (56.3%). Compared with MSSA, MRSA tended to have more symptoms, such as tachypnea, chest indrawing, contractions of the accessory respiratory muscles, crackles, wheezing, and hypoxemia (SpO_2_ ≤ 94%) in children with severe CAP. However, the difference was not statistically significant (except for signs of accessory respiratory muscle contraction, with *p* = 0.04). Therefore, there is insufficient evidence to determine that bacterial antibiotic resistance is associated with disease severity. Many recent studies have noted that there is no single symptom or sign that can be used to accurately diagnose pneumonia in children. In these studies, the specificity was improved when individual clinical factors such as tachypnea, fever, and hypoxemia were combined [28,29]. A decreased SpO_2_ value is useful for the diagnosis of pneumonia. Hypoxemia is a sign of serious illness and an indicator for hospitalization [13,30]. SpO_2_ ≤ 94% was considered the threshold to distinguish the severity of pneumonia, considering the indicator for hospitalization, in the study of Blanc J in Papua New Guinea [31]. This study also took the threshold SpO_2_ 94% for its analysis. As a result, 79.2% of children had peripheral blood oxygen saturation values of SpO_2_ ≤ 94%.

The mean WBC count of children with MRSA-related severe pneumonia was 13.56 ± 6.44 (×10^3^/mm^3^). Compared with other etiologies, MRSA was less likely to cause leukocytosis > 15,000/mm^3^ (*p* = 0.048). This proves that leukocytosis cannot be relied upon to assess the severity and predict the cause of the disease. Esposito S et al. highlighted that the leukocyte count had the lowest positive predictive value compared with procalcitonin and CRP [32]. This study recorded 58.1% of cases with an increase in CRP of >10 mg/L. In many studies, CRP has been implicated as an acute phase reactant associated with disease severity in pediatric bacterial infections [23]. However, there was no proper response to these data. It is worth emphasizing the need to assess the various elements as a whole, as individual elements are not decisive. Both WBC and CRP values are highly dependent on the timing of the test. For cases tested within a timeframe as short as that observed in this study (within the first 48 h after admission), the WBC count and CRP concentration may not be high.

### 4.2. Antibiotic Resistance and Minimum Inhibitory Concentration

The MRSA strains identified in this study were found to be resistant to many antibiotics, such as penicillin, clindamycin, erythromycin, and gentamicin. This result is similar to that of the study of Wu X et al. which found that 100% of MRSA strains were resistant to penicillin, 85.0% were resistant to erythromycin, and 67.7% were resistant to clindamycin [9]. Bacteria belonging to the MRSA strains are considered resistant to all β-lactam, including cephalosporins (except for ceftaroline and ceftobiprole); therefore, routine AST of staphylococci with β-lactams is not necessary. A report on multidrug-resistant (MDR) MRSA strains in Vietnam showed that up to 51.8% of MRSA isolates were MDR. This high antibiotic resistance may be explained by the relatively high rate of inappropriate antibiotic prescriptions in Vietnamese hospitals [33], and most antibiotics sold do not require a doctor’s prescription in many pharmacies [34].

Compared with MSSA, the MRSA strains were more resistant to clindamycin and erythromycin and less sensitive to ciprofloxacin and levofloxacin (*p* < 0.05). Therefore, these antibiotics should not be considered appropriate for the treatment of MRSA infections. This study found MRSA isolates to be fully sensitive to vancomycin, linezolid, and rifampin. However, while the efficacy of rifampin against *S. aureus* seems to be promising for the treatment of osteomyelitis or prosthetic device-related infections, it is not used as monotherapy because of its extremely high resistance [35]. Therefore, vancomycin and linezolid may be appropriate options for severe CAP caused by MRSA.

MRSA is classified as healthcare-associated methicillin-resistant *Staphylococcus aureus* (HA-MRSA) and community-associated methicillin-resistant *Staphylococcus aureus* (CA-MRSA) [2,36,37]. Compared to HA-MRSA, CA-MRSA is typically less resistant to antibiotics [2,36,37]. A study of 384 patients admitted to Mettu Karl Referral Hospital, South West Ethiopia, observed a high rate of isolation of MRSA and vancomycin-resistant *Staphylococcus aureus* (VRSA) in patients undergoing surgical intervention in hospital [38].

In this study, some antibiotics, such as penicillin, erythromycin, clindamycin, gentamicin, ciprofloxacin, levofloxacin, and chloramphenicol had an MIC_90_ equal to the resistance threshold according to the CLSI guidelines for 2021. Vancomycin, linezolid, and rifampin had an MIC_90_ of lower than the CLSI threshold of resistance, of which vancomycin had a 32-fold decreased MIC_90_ (0.5 mg/L), and linezolid had a 2-fold decreased MIC_90_ (4 mg/L). Thus, although up to 78.0% of MRSA strains were isolated through culture, the MICs of antibiotics (especially vancomycin) were mostly equal to or lower than the resistance threshold according to the CLSI. In some countries across the world, the MIC of vancomycin is of the same level (the MIC_90_ of vancomycin in the study of Doudoulakakis AG et al. in 2007–2014 was 2 mg/L) or much higher (the study of Ghahremani M et al. conducted on adults from 2012–2015 recorded 11.6% VRSA strains, including nine VRSA strains with an MIC for vancomycin of ≥256 mg/L) [19,39]. It is necessary to develop a rational antibiotic use strategy so as to prevent vancomycin resistance in the future. Empiric antibiotics can be used for mild pneumonia, but for severe pneumonia or initial antibiotic failures, they should be used with bacteriological confirmation.

One of the limitations of this research is its distinction between *S. aures* and other coagulase-positive *Staphylococci* strains (e.g., *S. schleiferi*, *S. intermedius*) by coagulase testing alone. However, it can be presumed that these agents, which have previously been recognized as veterinary pathogens commonly affecting domestic animals, have never been identified as pathogens in large animals [40,41]. If the isolates were to be confirmed simultaneously by a real-time polymerase chain reaction (PCR), a higher level of accuracy could be obtained. This is a direction for future research. Another limitation is that this study excluded children who had been hospitalized within the past 14 days instead of 30 days. It appears that this can lead to a conflation of CAP with possible healthcare-acquired pneumonia (HCAP). The basis for this decision was an old local guideline observed at the study site. Our further studies will be properly updated. Other limitations are that the study was conducted at a single institution, the effect of the small sample on the statistical analysis (especially analysis in Table 3), and the fact that there are more antibiotic agents that can treat MRSA beyond those included in this study, such as trimethoprim/sulfamethoxazole. This research will be carried out more comprehensively in further studies.

## 5. Conclusions

*S. aureus*, and mainly MRSA, was identified as the second leading cause of severe CAP in Vietnamese children. The MRSA strains were resistant to many antibiotics but fully sensitive to vancomycin and linezolid, with their MICs being well below the CLSI threshold in 2021. Therefore, vancomycin and linezolid may be appropriate options for severe CAP identified by MRSA.

## Figures and Tables

**Table 1:** Demographic, clinical, and subclinical characteristics.

Characteristics		Severe CAP Caused by MRSA (*n* = 32) *n*, (%)	Severe CAP Caused by MSSA (*n* = 9) *n*, (%)	Severe CAP Caused by Other Agents (*n* = 193) *n*, (%)	*p*1 Value	*p*2 Value
**Age**	Median, IQR (months)	14 (7–27)	10 (9–30)	17 (10–30)	0.603	0.306
	<2 years	24 (75.0)	5 (55.6)	116 (60.1)	0.433	0.436
	2–5 years	7 (21.9)	3 (33.3)	66 (34.2)		
	>5 years	1 (3.1)	1 (11.1)	11 (5.7)		
**Sex**	Male	25 (78.1)	5 (55.6)	119 (61.7)	0.177	0.164
**Symptoms and signs**	Fever	32 (100)	9 (100)	193 (100)	NA	NA
	Cough	32 (100)	9 (100)	193 (100)	NA	NA
	Vomiting	5 (15.6)	1(11.1)	24 (12.4)	0.735	0.702
	Diarrhea	4 (12.5)	2 (22.2)	32 (16.6)	0.439	0.819
	Tachypnea	30 (93.8)	7 (77.8)	172 (89.1)	0.154	0.832
	Chest indrawing	18 (56.3)	3 (33.3)	118 (61.1)	0.224	0.240
	Accessory muscle used	11 (34.4)	0	64 (33.2)	**0.04**	0.430
	Crackles	28 (87.5)	7 (77.8)	173 (89.6)	0.466	0.429
	Wheezing	16 (50.0)	2 (22.2)	114 (59.1)	0.138	0.075
	SpO_2_ ≤ 94%	19 (79.2) ^a^	4 (66.7) ^b^	106 (67.9) ^c^	0.517	0.343
**WBC count**	Mean ± SD (×10^3^/mm^3^)	13.56 ± 6.44	9.87 ± 5.14	14.33 ± 5.87	0.223	0.123
	>15,000/mm^3^	9 (28.1)	2 (22.2)	84 (43.5)	0.724	**0.048**
**CRP**	Median, IQR (mg/L)	12.5 (3.2–21.8)	12.3 (11.1–21.0)	12.5 (3.8–35.7)	0.897	0.567
	>10 mg/L	18 (58.1) ^a^	7 (77.8) ^b^	102 (55.1) ^c^	0.282	0.394

CAP: Community-acquired pneumonia; MRSA: Methicillin-resistant *Staphylococcus aureus*; MSSA: Methicillin-sensitive *Staphylococcus aureus*; CRP: C-reactive protein; IQR: Interquartile Range; SD: Standard Deviation. ^a^: n < 32, ^b^: n < 9, ^c^: n < 193, omission error due to not being collected upon admission by the clinician or administration of procalcitonin to replace CRP. *p*1: Comparison between 2 groups of severe CAP due to MRSA and MSSA. *p*2: Comparison between 2 groups of severe CAP caused by MRSA and other agents. NA: Not Applicable.

**Table 2:** The susceptibility to antibiotics of MSSA (*n* = 9) and MRSA (*n* = 32).

Antibiotics	MSSA (*n* = 9)			MRSA (*n* = 32)			*p*
	S *n* (%)	I *n* (%)	R *n* (%)	S *n* (%)	I *n* (%)	R *n* (%)	
Penicillin	1 (11.1)	0	8 (88.9)	0	0	32 (100)	0.056
Clindamycin	5 (55.6)	1 (11.1)	3 (33.3)	4 (12.5)	1 (3.1)	27 (84.4)	**0.009**
Erythromycin	6 (66.7)	0	3 (33.3)	4 (12.5)	3 (9.4)	25 (78.1)	**0.003**
Gentamicin	4 (44.4)	0	5 (55.6)	14 (43.8)	0	18 (56.3)	0.970
Ciprofloxacin	8 (88.9)	1 (11.1)	0	21 (65.6)	0	11 (34.4)	**0.028**
Levofloxacin	9 (100)	0	0	21 (65.6)	0	11 (34.4)	**0.040**
Chloramphenicol	8 (88.9)	0	1 (11.1)	28 (87.5)	0	4 (12.5)	0.910
Vancomycin	9 (100)	0	0	32 (100)	0	0	NA
Linezolid	9 (100)	0	0	32 (100)	0	0	NA
Rifampin	9 (100)	0	0	32 (100)	0	0	NA

S: Susceptible; I: Intermediate; R: Resistant. NA: Not Applicable. MSSA: Methicillin-sensitive *Staphylococcus aureus.* MRSA: Methicillin-resistant *Staphylococcus aureus.*

**Table 3:** MIC distribution of antibiotics for isolated *S. aureus* (n = 41).

Antibiotics	Number of Isolates at MIC Values (mg/L)												
	0.12	0.18	0.25	0.38	0.5	0.75	1	2	4	5	8	16	32
Penicillin	2		**39**										
Clindamycin					9		2		**20**	8	2		
Erythromycin					10		3				**28**		
Gentamicin									18			**23**	
Ciprofloxacin					2		27	1	4			**7**	
Levofloxacin							30		**11**				
Chloramphenicol											36		**5**
Vancomycin		2	15	11	**9**	**4**							
Linezolid									**41**				
Rifampin							**41**						

MIC: minimal inhibitory concentration. Results in bold are the frequencies for the values of MIC_90_, the concentration at which 90% of bacterial strains are inhibited.

## Data Availability

The datasets generated and/or analyzed in this study are not publicly available because they are the property of Can Tho University of Medicine and Pharmacy and Hanoi Medical University; however, they may be available from the corresponding author upon reasonable request.
